# A Bibliometric Analysis of the Top 30 Most-cited Articles in Gestational Diabetes Mellitus Literature (1946-2019)

**DOI:** 10.7759/cureus.4131

**Published:** 2019-02-25

**Authors:** Pulwasha M Iftikhar, Fatima Ali, Mohammed Faisaluddin, Azadeh Khayyat, Maribel De Gouvia De Sa, Tanushree Rao

**Affiliations:** 1 Obstetrics and Gynecology, St. John's University, New York, USA; 2 Obstetrics and Gynecology, Shifa International Hospital, Islamabad, PAK; 3 Obstetrics and Gynecology, Deccan College of Medical Sciences, Hyderabad, IND; 4 Internal Medicine, Ahvaz Jundishapur University of Medical Sciences, Ahvaz, IRN; 5 Obstetrics and Gynecology, Aberdeen Royal Infirmary, Aberdeen, GBR; 6 Obstetrics and Gynecology, Liverpool Hospital, New South Wales, AUS

**Keywords:** gestational diabetes mellitus, bibliometrics, web of science, citation classics, impact factor, obstetrics, research, citations, scopus

## Abstract

Objective

The aim of this bibliometric analysis is to evaluate the importance and impact of the articles that have been published with the title gestational diabetes mellitus (GDM) in the specialty of obstetrics & gynecology and endocrinology during the period 1946-2019. It also reveals that the area of GDM has received increased attention and interest by researchers, research funding institutions, and practitioners.

Material and methods

A thorough database search of Scopus and Web of Science was performed and the articles pertaining to gestational diabetes mellitus that were published between 1946 and 2019 were reviewed by two reviewers, Iftikhar PM and Ali F, with respect to their year of publication, authors, country of origin, journal of publication, and the affiliated institutions of the authors as well as journals. Institutional review board approval was not required for this study, as the data being analyzed were already available electronically, and otherwise, in libraries and databases.

Results

The 30 most-cited articles on gestational diabetes mellitus were thoroughly analyzed. The top article was cited 5028 times while the least number of citations for any article was 328. Among these 30 articles, five were published in the year 2005, which is the highest number of publications in any given year of the timeline being considered in this study. Most of the articles (n = 18) were from the United States of America, followed by Australia (n = 3); other countries contributed to two or fewer articles. Diabetes Care made most (n = 8) of the list. We found one author who had three publications and the rest contributed two or less articles. The top article in our study was cited almost 5028 times; meanwhile, there are 13 journals from different specialties that have referenced the most cited articles pertaining to gestational diabetes.

Conclusion

Our bibliometric analysis provides a picture of scientific research, which will help in evidence-based descriptions, comparisons, and visualizations of research output in GDM, and it can be used to explicate and describe the patterns of performance and impact of GDM research.

## Introduction

Gestational diabetes mellitus (GDM) is defined as the onset of glucose intolerance during pregnancy. The incidence and prevalence of GDM have been on the rise in recent years, currently affecting 6%-8% percent of all pregnancies. It is one of the most studied subject areas in the specialties of obstetrics and gynecology, pediatrics, and endocrinology.

Bibliometric analysis is defined as a statistical evaluation of published scientific articles, books, or the chapters of a book, and it is an effectual way to measure the influence of publication in the scientific community [1-4]. The academic impact of a piece of research can be gauged by the number of times it has been cited by other authors [5-7]. The study design of a bibliometric analysis or citation classics is a widely used technique to assess the impact of an article [1,8-9]. The determination of a citation hierarchy list in one specialty of the medical field, formed by numerous journals that are specific to one specialty, is a process that requires more time and expertise as compared to the bibliometric analysis of just one journal [10-11].

Research in GDM has expanded significantly over the past four decades. As witnessed in other fields of medicine, the trend and progression of research in obstetrics and gynecology has been compiled by various publications and studies carried out by peers. The evolution of this research led to the introduction and implementation of the concept of evidence-based medicine [3]. Publications of prime medical journals pertaining to the field of obstetrics and gynecology started almost over a century ago, with the American Journal of Obstetrics and Gynecology and Obstetrics and Gynecology being among the top few. Researchers and doctors working in the field of obstetrics and gynecology have worked with over 180 journals, which have been listed in the Scopus Database Library as well in Web of Science, to publish their works in [6-8]. The aforementioned journals do not just cover the subspecialties of obstetrics and gynecology but also cover specific subject matters, such as gestational diabetes mellitus (like Gynecological Endocrinology), as will be discussed in this paper.

Authors will quote the articles in their peer-reviewed studies that already have some literary significance, which is a measure of the number of times that article has been cited before [10-12]. It is worth noting here that the number of times an article has been referenced is not directly proportional to the quality of the study, and not even the impact it has had on the clinical or medical practices of the author himself or of his colleagues. However, it does have the potential to influence readership of the particular subject and, therefore, leads to changes in discussions, practice essentials, controversies, and even further research in the specialty or the subject [10]. As stated above, a citation classic in specific subject matters and journals of specific specialties has now become one of the most popular research methodologies when it comes to deducing the research impact of an article, journal, or its author [13].

Referencing and other literary impact data collection was started in 1945 by the Institute for Scientific Information (ISI) and is still ongoing. This information was electronically made available in 1979. Science Citation Index Expanded is the most recent journal citation system and database that has been made available by Web of Science [3,12-14]. Almost 10,000 journals with a high-impact index are available in this database, from all fields, including social sciences, humanities, and arts. The objective behind the evaluation of the features of these articles was to determine the importance of these articles with respect to the subject of gestational diabetes mellitus and the specialty of obstetrics and gynecology. Changes in the level of evidence presented in these articles with respect to time have also been studied.

## Materials and methods

A thorough database search of Google Scholar, Scopus, and Web of Science was conducted to retrieve and analyze the most cited 30 articles in gestational diabetes mellitus literature from 1946 till January 2019 by two independent reviewers, Iftikhar PM and Ali F, using the option “cited reference search” for identifying the citations published under the topic of gestational diabetes mellitus that belonged to different specialties, including obstetrics and gynecology, endocrinology, and physiology [15-17]. The list of the 30 most-cited articles is given in the Appendix.

For this study, we have retrieved and evaluated the electronically available data retrospectively, so institutional review board approval was not required. All the articles and journals that were made part of the study were in the English language. It is worth noting that in our study, we did not limit our research of the most cited 30 articles to journals from the obstetrics and gynecology genre. Clinical articles from various journals from across the world and journals of medicine, pediatrics, endocrinology, and even physiology were studied through, in order to make sure that none of the articles pertaining to gestational diabetes mellitus were missed out.

There were many telescopes that were used to view and analyze each one of the 30 articles that were shortlisted as the most cited ones. The articles were reviewed from the aspect of source journal, author, the geographic placement of the authors, institution, and year of publication. Typing and subtyping of these 30 articles was also done, as they were categorized as either clinical research or basic science articles, and, furthermore, as prospective cohort studies, review articles, case reports, case series, expert opinions, randomized-controlled clinical trial, basic science-animal research, basic science biomechanics, or basic science in vitro studies.

Journal citation reports were made use of in order to quantitatively analyze and evaluate whether the top 30 articles that are being reviewed for this study were included in the Science Citation Index (SCI) or the Science Citation Index Expanded. The impact factor related to the publication year was also obtained.

## Results

**Figure 1:**
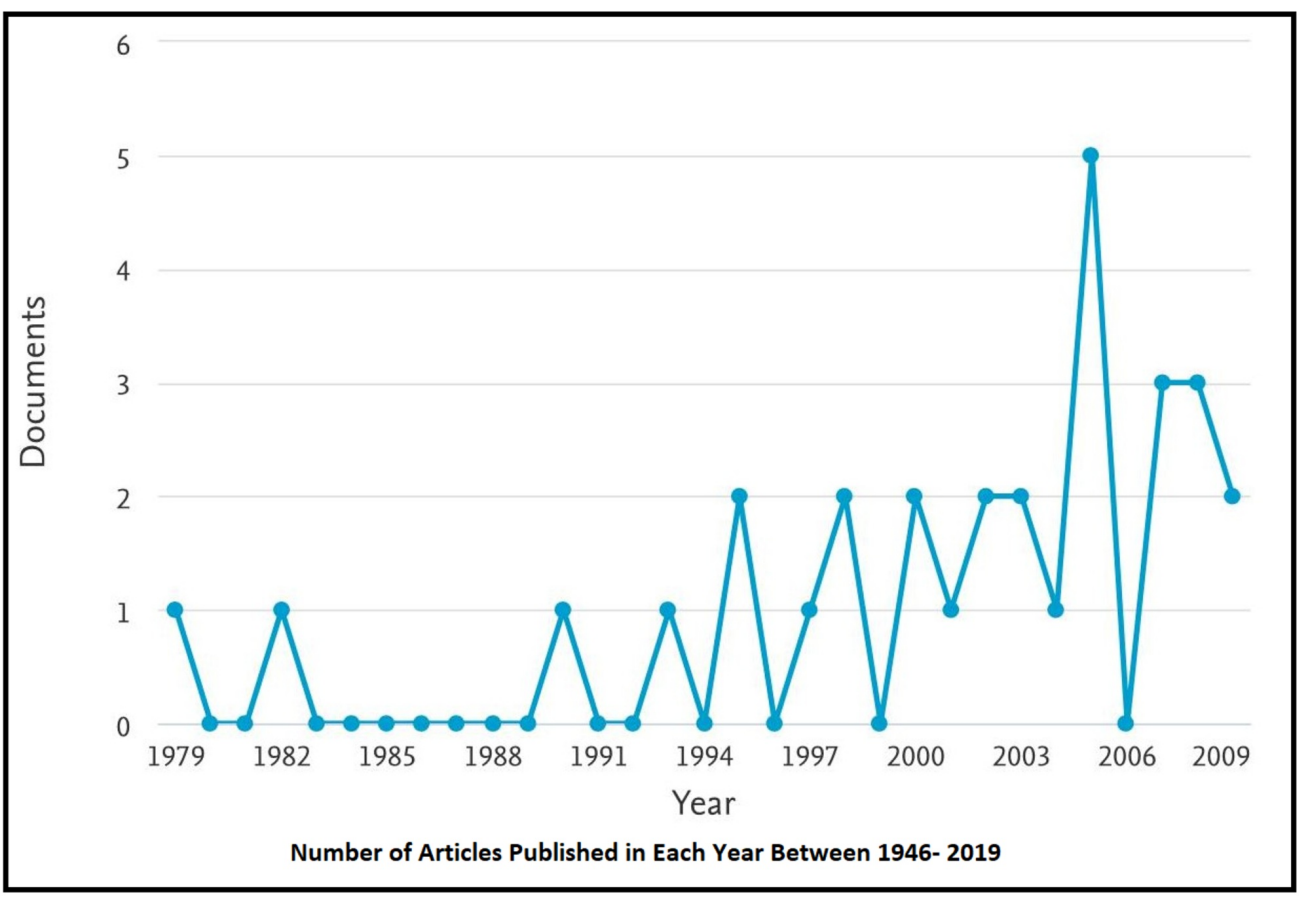
Number of Articles Published Each Year Between 1946 and 2019

The span of citations of the most referenced 30 articles ranged from 328 to 5028 times; meanwhile, the top-five cited articles were used over 1000 times by authors of the fraternity. As for the time span of the publication years of the aforementioned articles, it ranged from 1979 to 2009. The year in which the most number of articles from among the top 30 articles were published was 2005, as the five most referenced articles were published in this year alone (Figure 1). The language of publication of all the aforementioned articles was English.

**Figure 2:**
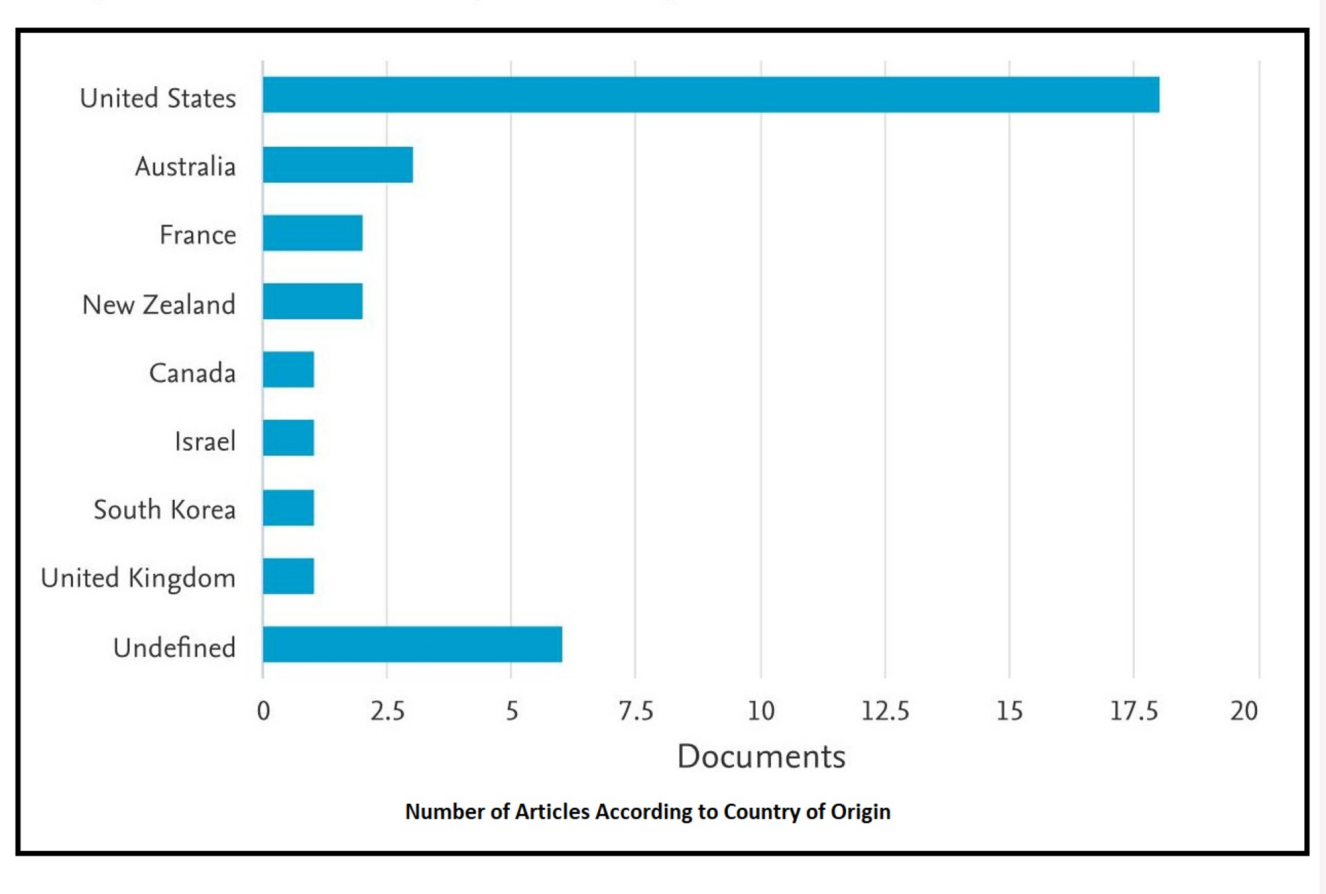
Number of Articles According to Country of Origin

The list of the top-30 most-cited articles that was comprised after searching Scopus and Web of Science included articles from across the world; nevertheless, the greatest number of articles was published in the United States (n = 18), followed by Australia (n=3), France (n=2), and New Zealand (n=2). Other countries of origin included Israel, Canada, the United Kingdom, and South Korea (Figure 2). The authors and their credentials are one of the top features analyzed in Citations Classics. For this study, the authors that bagged the most articles in the top 30 articles were Metzger BE (n = 3) followed by Buchanan TA (n = 2), Catalano PM (n = 2), and Carpenter MW (n = 2). Amongst the top 10 authors for this study of citation classics were Colditz GA, Dablea D, Langer O, Xenakis EMJ, and Yogev Y.

**Figure 3:**
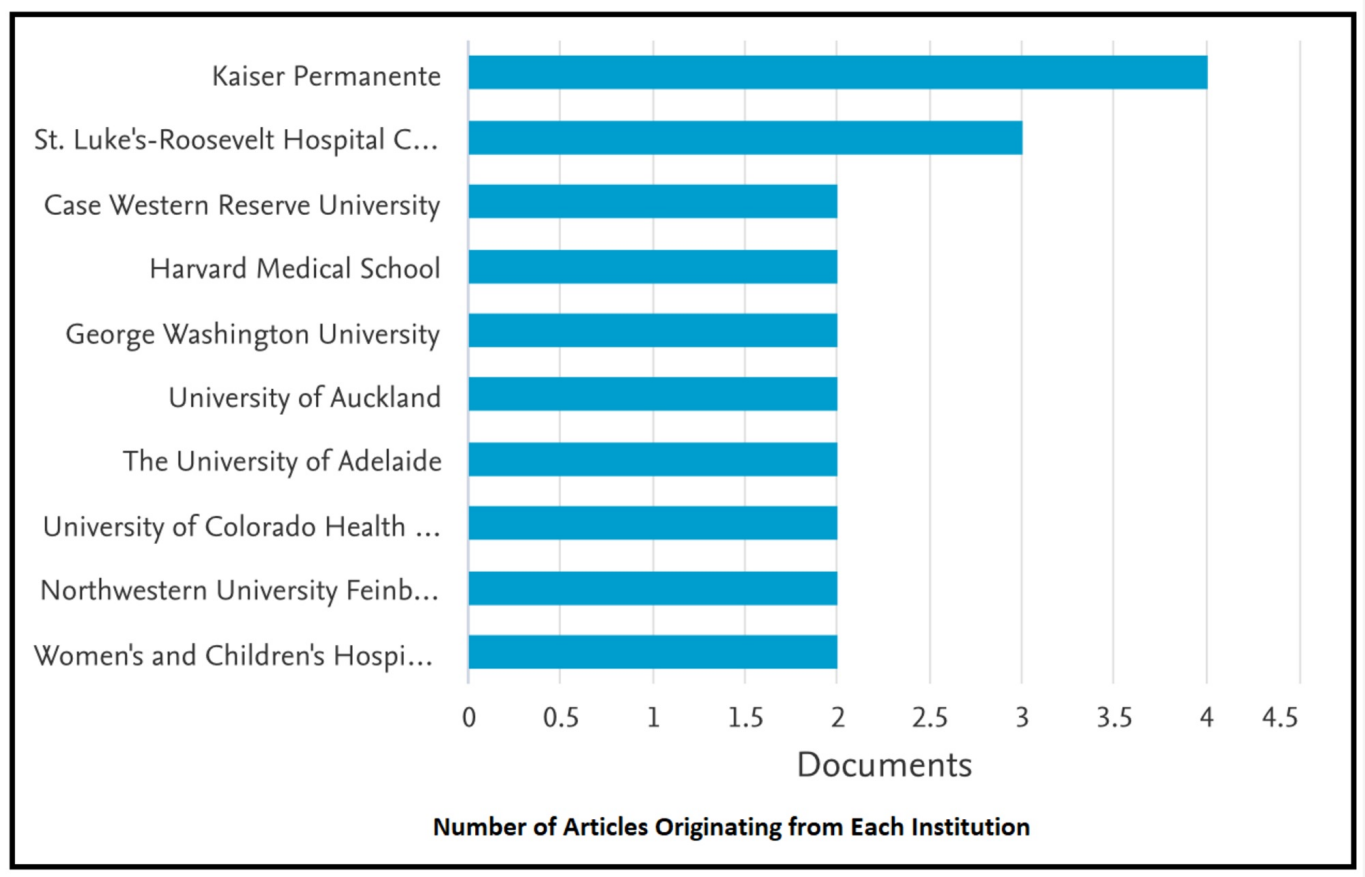
Number of Articles Originating from Each Institution

The institutions with which the top-30 cited articles included in our study are associated are Kaiser Permanente, St. Luke Roosevelt Hospital Center, Case Western Reserve University, Harvard Medical School, George Washington University, University of Auckland, and the University of Adelaide (Figure 3).

The majority of the articles that were included in the top-30 cited articles for gestational diabetes mellitus were primarily clinical research as well as basic science research articles (n = 46). The subtypes of these articles included prospective cohorts, case reports, case series, cross-sectional studies, randomized controlled trials, animal studies, and biochemical and in vitro studies. Other publications in this study included 10% conference papers (n = 3) and 16.7% review articles (n = 5). The journals in which these top-30 articles were cited stemmed from various genres, including medicine, obstetrics and gynecology, and other specialties: 73.2% (n=30), nursing: 19.5% (n=8), biochemistry, genetics, and molecular biology: 7.3% (n = 3).

After thoroughly analyzing the articles and establishing the level of evidence, the impact factor for the journals that were included in the study was determined. The top five journals in which these articles were published included Diabetes Care (n = 8), American Journal of Obstetrics and Gynecology (n = 4), Diabetes (n = 2), New England Journal of Medicine (n = 5), and Pediatrics (n = 2). The most cited GDM articles were found in 13 journals (Table 1).

**Table 1:** Journals with Highest-cited Gestational Diabetes Mellitus and Their Impact Factor

List of Journals with Highest-cited Gestational Diabetes Mellitus Articles and Their Impact Factors			
No.	Journals	Impact Factor	No. of articles
1	Diabetic Care	13.397	6
2	American Journal of Obstetrics and Gynecology	5.732	4
3	New England Journal of Medicine	79.258	6
4	Diabetes	7.273	4
5	Journal of American Medical Association	47.661	1
6	Lancet	53.254	1
7	Pediatrics	5.515	2
8	Journal of Clinical Endocrinology And Metabolism	5.789	1
9	British Journal of Obstetrics and Gynecology	5.051	1
10	Journal of Clinical Investigation	13.251	1
11	International Journal of Obesity	5.337	1
12	American Journal of Clinical Nutrition	3.41	1
13	Diabetic Medicine	3.054	1

## Discussion

Gestational diabetes mellitus is one of the most studied topics in the specialty of obstetrics and gynecology. What is interesting to note about this topic is that it is not just confined to this specialty but also comes under the banner of endocrinology and general medicine. Moreover, some physiology journals also have discussed the topic extensively. The topic's importance is evident from the fact that gestational diabetes not only has long-term effects on the mother but also impacts the health of the child. It will not be wrong to say that over time, the trend of research in the specialty of obstetrics and gynecology has been relocated towards gestational diabetes mellitus as one of the most studied subject matters. The top-30 articles and their associated citations are shown in the Appendix (Table 2).

It is important to note that the analysis and evaluation of scientific output from this citation classics study include the implementation of clinical research to clinical and medical practice. It is a common observation that the knowledge of clinicians pertaining to published research is seen to be either incomplete or insufficient because research findings are not presented in a systematic format, as they are dispersed among various journals, which makes it difficult to interpret the published scientific evidence. Bibliometric analyses often address the aforementioned issues by playing the role of a systematized amalgamation of all kinds of clinical research done in a certain field or subjective area [15-16].

A bibliometric analysis represents an understanding that provides a cross-sectional view and the current state of research work on the topic of interest. It is a statistical and quantitative analysis that aims at identifying the scholarly impact and characteristics of publications within a specific research field, which could provide useful information to researchers involved in the development of research strategies to address the health issues. Many scholars have investigated the most cited articles that describe the advances in various specialties and subspecialties. This study will help recognize the quality of the work, discoveries, and trends pertaining to gestational diabetes mellitus. Many clinical and basic research papers have reported the potential adverse effects in neonates due to gestational diabetes mellitus, including macrosomia, fetal death, shoulder dystocia, nerve palsy, hypoglycemia, and respiratory distress. Lee et al. reported a cumulative risk of 25.8% at 15 years post-pregnancy in moderate-risk patients [18]. The overall incidence of gestational diabetes mellitus is as high as 70% in some populations. The diagnosis and effective management of gestational diabetes mellitus improve maternal and fetal outcomes. The early diagnosis of "diabetes in pregnancy" needs prompt evaluation and treatment [18]. Therefore, this analytic study will enable academic institutions and funding agencies to assess the research quality and productivity of individual researchers. Moreover, the high interest in this field suggests expanded research opportunities in the future [19].

Like any other research methodology, a citation classic has both intrinsic limitations and strengths. The fact that most of the articles that made it to the top-30 most-cited papers originated from the United States and the United Kingdom is reflective of the observation that either most clinical trials are carried out in the developed world or that articles published in the English language and international journals are the ones that are widely cited. For instance, citation analysis of research productivity in emergency medicine physicians and researchers of South Korea shows that although the researchers were placed in Korea and the potential impact of the study was also bound to take place in South Korea, the researchers preferred to have their publications in international English language journals. Furthermore, research articles found on Korea Med in the time span between 1992 and 2015 were not included in this bibliometric analysis [15].

In another citation classic pertaining to emergency medicine, carried out by Wilson and Itagaki, it was concluded that almost 14,000 publications in the specialty of emergency medicine between the years 1996 and 2005 were authored by researchers that were geographically placed and affiliated with departments of emergency medicine in the United States, as 58.5% of the articles were from institutions based in the US, followed by the UK (8.4%) [20]. These results are comparable to our citation classic, as more than 50% of the articles included in the study were from the United States [21-22].

As has been discussed earlier in this paper, the number of citations is seen to be directly proportional to the significance of the particular publication in the relevant specialty, so it is important for us to analyze and compare the number of citations of the top-cited articles of our study with other studies. In the citation analysis conducted in a department of emergency medicine in South Korea, the number of times an article was most cited in Web of Science was 85. Meanwhile, the gestational diabetes mellitus article that was most cited in our bibliometric analysis was referenced 4877 times. As made evident by statistics, the number of citations of our top-cited article is considered to be significantly higher and parallel to research in other fields. Citation counts can also differ due to the number of aggregated articles varying with different search engines since Google Scholar and Scopus have been shown to retrieve more quotations for a single article as compared to Web of Science [23-24].

The quantitative analysis of our study was performed with the help of the impact factor of the journals that were being considered. It is, therefore, imperative to bring into the limelight the significance of the impact factor, the term that was first coined in the year 1963. For instance, if the Journal Citation Reports states a journal has an impact factor of 2, it implies that the research published in that particular journal during the previous two years were approximately referenced two times in journals that are SCI-indexed during the following year. As a matter of fact, the purpose of the impact factor is the evaluation of journals and not the evaluation of individual authors and articles that have been published in that journal. In our study, the highest impact factor scores were assigned to articles that were published in the New England Journal of Medicine, Journal of American Medical Association, and Lancet, which is comparable to the South Korean study in which the top impact factor scores were of articles from the New England Journal of Medicine, Lancet, and Circulation [15], as shown in the Appendix.

By analyzing this bibliometric study, it can be concluded that gestational diabetes mellitus is a subject that is extensively researched. However, it is hard to conclude the trend of this research since the top-five most-cited articles were related to all aspects of gestational diabetes mellitus, including the diagnosis, management, and impact of the disease on the mothers’ and infants’ health.

Even though the data analysis of this study was rather objective and comprehensive, the presence of limitations cannot be excluded. As stated above, the first and foremost limitation pertains to the articles included in the study being only in the English language and from the developed world. Second, the citation classics study design is limited by time. For example, if a high-quality research article on gestational diabetes mellitus has been published in recent years, we cannot expect it to be cited more than an article that has been around for a decade or more.

## Conclusions

Our bibliometric analysis provides both quantitative and qualitative analyses of the most cited articles in gestational diabetes. It also provides insights into scientific research, which will assist in generating evidence-based descriptions, comparisons, and visualizations of research output in gestational diabetes mellitus. It can be used to explicate and describe patterns of performance and the impact of gestational diabetes mellitus research.

## Appendices

**Table 2:** Top 30 Most-cited Articles in Gestational Diabetes Literature

	Most-cited 30 articles in Gestational Diabetes Mellitus			
	Title	Journals	Reference No	No. of Citations
1	Classification and diagnosis of diabetes mellitus and other categories of glucose intolerance	Diabetes	1979;28:1039-57	5028
2	Hyperglycemia and adverse pregnancy outcome	N Eng J Med	2008;358:191-02	2188
3	Effect of treatment of gestational diabetes mellitus on pregnancy outcomes	N Eng J Med	2005;352:2477-86	1727
4	Metabolic syndrome in childhood: Association with birth weight, maternal obesity, and gestational diabetes mellitus	Pediatrics	2005;115:290-96	1326
5	A multicenter, randomized trial of treatment for mild gestational diabetes.	N Eng J Med	2009;361:1339-48	1202
6	Gestational diabetes and the incidence of type 2 diabetes - a systematic review	Lancet	2009;373:1773-79	1187
7	Criteria for screening-test for gestational diabetes	Am J Obstet Gynecol	1981;144:768-73	1170
8	Maternal obesity and pregnancy outcome: a study of 287 213 pregnancies in London	Diabetes Care	2002;25:1862-68	1139
9	Type 2 diabetes mellitus after gestational diabetes: a systematic review and meta-analysis	Int J Obesity	2001;25:1175-82	981
10	A comparison of glyburide and insulin in women with gestational diabetes mellitus	N Eng J Med	2000;343:1134-38	575
11	Metformin versus insulin for the treatment of gestational diabetes	N Eng J Med	2008;358:2003-15	555
12	Gestational diabetes mellitus	J Clin Invest	2005;115:485-91	498
13	Increasing prevalence of gestational diabetes mellitus: a public health prospective	Diabetes Care	2007;30:141-46	496
14	Increasing prevalence of gestational diabetes mellitus (GDM) over time and by birth cohort - Kaiser Permanente of Colorado GDM screening program	Diabetes Care	2005;28:579-84	465
15	Epidemiology of gestational diabetes mellitus and its association with type 2 diabetes	Diabetic Medicine	2004;21:103-13	463
16	Postprandial versus preprandial blood-glucose monitoring in woman with gestational diabetes-mellitus requiring insulin therapy	N Eng J Med	1995;333:1237-41	461
17	A prospective study of pregravid determinants of gestational diabetes mellitus	JAMA	1991;278:1078-83	456
18	Maternal obesity and risk of gestational diabetes mellitus	Diabetes Care	2007;30:2070-76	445
19	Maternal gestational diabetes, birth weight, and adolescent obesity	Pediatrics	2003;111:2096:	427
20	The short- and long-term implications of maternal obesity on the mother and her offspring	BJOG	2006;113:1126-33	425
21	Gestational diabetes: The consequences of not treating	Am J Obstet Gynecol	2005;192:989-97	405
22	TNF-α is a predictor of insulin resistance in human pregnancy	Diabetes	2002;51:2007-18	400
23	Long term effects of the intrauterine environment: The Northwestern University diabetes in pregnancy center	Diabetes Care	1998;21:142-49	395
24	Childhood obesity and metabolic imprinting: The ongoing effects of maternal hyperglycemia	Diabetes Care	2007;30:2287-92	391
25	Prevention of diabetes in women with a history of gestational diabetes: effects of metformin and lifestyle Interventions	J Clin Endocrinol Metab	2008;93:4774-79	364
26	Carbohydrate and lipid metabolism in pregnancy: normal compared with gestational diabetes mellitus	Am J Clinical Nutrition	2000;71:1256-61	353
27	Trends in the prevalence of preexisting diabetes and gestational diabetes mellitus among a racially/ethnically diverse population of pregnant women, 1999-2005	Diabetes	2008;31:899-04	352
28	The influence of obesity and diabetes on the prevalence of macrosomia	Am J Obstet Gynecol	2004;191:964-68	340
29	Insulin sensitivity and B cell responsiveness to glucose during late pregnancy and moderately obese women with normal glucose tolerance or mild gestational diabetes	Am J Obstet Gynecol	1990;162:1008-14	330
30	Hyperglycemia and Adverse Pregnancy Outcome (HAPO) Study Associations With Neonatal Anthropometrics	Diabetes	2009;58:453-59	328
